# A Japanese patient with ductal carcinoma of the prostate carrying an adenomatosis polyposis coli gene mutation: a case report

**DOI:** 10.1186/s13000-020-01016-9

**Published:** 2020-08-06

**Authors:** Kota Umeda, Takeo Kosaka, Kohei Nakamura, Toshikazu Takeda, Shuji Mikami, Hiroshi Nishihara, Mototsugu Oya

**Affiliations:** 1 Department of Urology, Keio University School of Medicine, 35 Shinanomachi, Shinjuku-ku, Tokyo, 160-8582 Japan; 2 Genomics Unit, Keio Cancer Center, Keio University School of Medicine, 35 Shinanomachi, Shinjuku-ku, Tokyo, 160-8582 Japan; 3 Division of Diagnostic Pathology, Keio University Hospital, 35 Shinanomachi, Shinjuku-ku, Tokyo, 160-8582 Japan

**Keywords:** Ductal carcinoma of the prostate, Prostatic neoplasms, Beta catenin, Genomic profiling, Wnt signaling pathway

## Abstract

**Background:**

Ductal carcinoma of the prostate is a histological subtype with a higher mortality than acinar adenocarcinoma. The number of cases is small and there are no treatment guidelines. We believe that this is the first report of ductal carcinoma of the prostate with an adenomatosis polyposis coli (*APC*) gene mutation in Japan.

**Case presentation:**

An 85-year-old man presented with gross hematuria, and a papillary tumor in the prostatic urethra that was diagnosed as ductal carcinoma of the prostate following transurethral resection. Genetic analysis found an APC mutation with loss of heterozygosity. Immunostaining revealed focal nuclear translocation of β-catenin. APC mutations associated with loss of β-catenin degradation in the Wnt signaling pathway and result in over accumulation of β-catenin are thought to increase mortality. In this patient, β-catenin migrated into tumor cell nuclei.

**Conclusion:**

To the best of our knowledge, this is the first report of ductal carcinoma of the prostate with an APC mutation in Japan. The development of a therapeutic Wnt inhibitor is discussed.

## Introduction

Ductal carcinoma of the prostate is a histologic subtype that was first described in 1967. It has an estimated incidence of 0.5 to 6% of all diagnosed prostate cancers and clinical implications that are not well understood [1, 2]. Approximately 30% of men with ductal carcinoma of the prostate present with clinical stage T3 or more advanced disease compared with (7%) of those with acinar carcinoma. The prostate-specific mortality of ductal carcinoma is significantly worse than that of acinar carcinoma [2]. Because ductal carcinoma of the prostate is relatively rare, treatment guidelines have not been established. The accumulation of case reports and genetic analysis are expected to add to the understanding and guide treatment of this type of prostate cancer. This patient is an 85-year-old male with ductal carcinoma of the prostate that carried an adenomatosis polyposis coli gene mutation.

## Case presentation

**Fig. 1:**
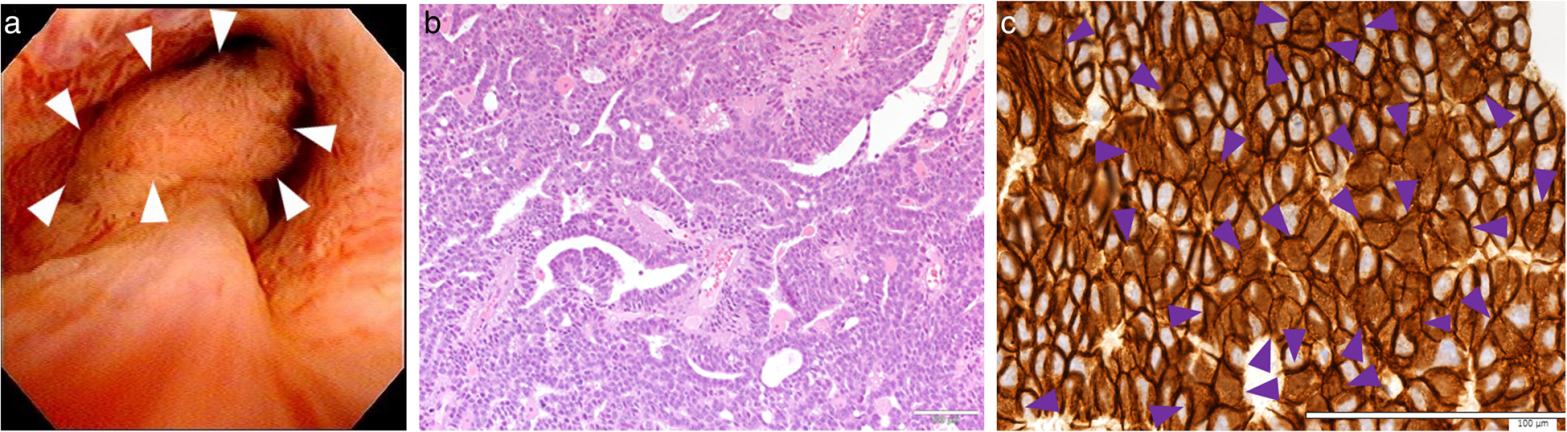
**a** Tumor in the urethra with representative images of hematoxylin and eosin (HE) (**b**) and β-catenin (**c**) staining. HE staining showed papillary architecture with morphological features of ductal adenocarcinoma including tall, columnar, pseudostratified epithelium. Focal nuclear β-catenin staining is seen (arrows in c). Bars indicate 100 μm

**Fig. 2:**
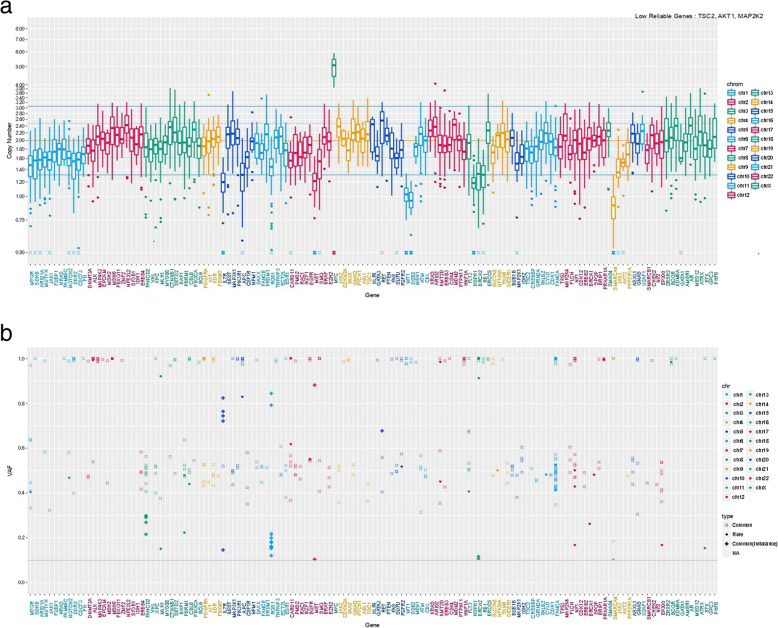
Results of genetic analysis with (**a**) genes shown on the horizontal axis and copy number on the vertical axis and (**b**) genes on the horizontal axis and variant allele frequency on the vertical axis

An 85-year-old man presented with chronic renal failure and gross hematuria. Cystoscopy revealed a papillary tumor in the prostatic urethra (Fig. 1a). His prostate-specific antigen was 1.06 ng/mL. We suspected urothelial carcinoma, but after performing transurethral resection of bladder tumor, the pathological diagnosis was ductal carcinoma of the prostate because of tall, pseudostratified columnar epithelium with abundant cytoplasm in a cribriform-papillary pattern of the resected tissue (Fig. 1b). Computed tomography and magnetic resonance imaging showed no evidence of primary or metastatic lesions. Genomic DNA sequencing of tumor tissue was performed with an average depth of 348.9×. The average tumor cellularity was 80% by both histological evaluation and variant allele frequency. Actionable gene alterations included a somatic frameshift *APC* (p.T1556Nfs*3) mutation with loss of heterozygosity (LOH), variant allele frequency of 83%, and a copy number of 1.1 (Fig. 2). *MYC* amplification was observed with a copy number of six. LOH without mutation was observed in SMARCA4 and WT1. Immunostaining showed diffuse membranous and partial nuclear β-catenin (Fig. 1c). He was treated with intensity-modulated radiation therapy consisting of 70 Gy administered in 28 fractions. There has been no recurrence or metastasis 1 year after the surgery (Table 1).

**Table 1:** Timeline

December 2018	Gross hematuria
March 2019	Cystoscopy, PSA 1.06
April 2019	TURBT
May 2019	IMRT 70Gy/28Fr
April 2020	No recurrence or metastases

## Discussion

### APC and the Wnt signaling pathway

*APC* is a tumor suppressor gene. Mutations are known to cause familial adenomatous polyposis (FAP), and are found in more than 80% of sporadic colorectal tumors [3]. *APC* gene mutations were first reported in FAP in 1991, and the progression from adenoma to cancer is thought to involve multistage carcinogenesis with accumulation of *APC*, *KRAS* and *TP53* gene mutations [3, 4]. WNT is a secreted glycoprotein that mediates cell proliferation, differentiation, motility, and polarity during embryonic and organ development [5]. β-catenin is a key effector in the Wnt signaling pathway, which controls cytoskeleton activity and cell movement via both β-catenin-dependent and -independent pathways. Wnt binds to seven transmembrane receptor (frizzled) proteins and single transmembrane coreceptors (LRP5, LRP6, ROR2 and RYK) [6]. APC protein promotes β-catenin degradation by binding to axin and as well as directly binding to β-catenin. Mutant APC binds to β-catenin but not to axin, which results in inefficient phosphorylation, incomplete degradation, and accumulation of β-catenin. In this patient, β-catenin was transferred to the nucleus (Fig. 1c). A frameshift mutation with LOH resulted in inactivation of both APC alleles and loss of *APC* gene function. We believe that this APC mutation affected Wnt signal transduction.

### Wnt signaling pathway in prostate cancer

Wnt signaling was shown to be involved in the progression of prostatic intraepithelial neoplasia (PIN) to prostate adenocarcinoma in mouse prostate cancer models and to induce high-grade PIN in differentiated luminal cells [5]. Abnormal immunoexpression of β-catenin in prostate cancer cells was found to increase the risk of death from tumor progression, and changes in immunohistochemical staining of β-catenin associated with high Gleason scores may have prognostic value. β-catenin overexpression has been linked to prostate cancer progression, high-grade intraepithelial neoplasia, and resistance to castration in several genetically engineered mouse models [5]. Genetic analysis studies have found APC mutations in 4% of patients with locoregional prostate cancer, that APC alterations were enriched in metastatic (15%) versus locoregional disease, and that 24% of patients with ductal carcinoma of the prostate had APC mutations [7, 8]. In this case, mutation was found in APC at p.T1556Nfs*3. In previous reports, mutations were not in the same place, but a high incidence of this mutation is characteristic of ductal carcinoma [8]. Ductal carcinoma of the prostate with an APC mutation has not previously been reported in Japan. In this patient, the APC loss-of-function mutation was accompanied by translocation of β-catenin into the nucleus, and may have had a poor prognosis.

### Targeting the Wnt signaling pathway

Anticancer drugs that block the Wnt signaling pathway are not currently available, but some are in development. OMP-18R5 (Vantictumab) targets the FZD1, FZD2, FZD5, FZD7, and FZD8 frizzled protein receptors to block WNT signaling are being investigated in phase I clinical trials in breast, pancreatic, and non-small cell lung cancer. LGK974, which blocks WNT signaling by targeting porcupine, is currently in phase I and II clinical trials in colorectal cancer [9]. No effective treatment that targets APC mutation are under investigation, but clinical trials of Wnt inhibitors such as vantictumab and LGK974 are expected.

## Conclusions

To the best of our knowledge, this is the first report of ductal carcinoma of the prostate with an APC mutation in Japan. Genetic analysis may be help to guide the development of candidate targeted drugs for ductal carcinoma of the prostate.

## Data Availability

Not applicable.

## Abbreviations

APC: Adenomatosis polyposis coli
FAP: Familial adenomatous polyposis
HE: Hematoxylin and eosin
LOH: Loss of heterozygosity
PIN: Prostatic intraepithelial neoplasia
